# Reproductive Factors and Breast Cancer Risk in Lithuanian Women: A Population-Based Cohort Study

**DOI:** 10.15388/Amed.2020.27.2.4

**Published:** 2020-12-21

**Authors:** Laura Steponavičienė, Rasa Vansevičiūtė, Lina Zabulienė, Domantas Jasilionis, Vincas Urbonas, Giedrė Smailytė

**Affiliations:** Laboratory of Cancer Epidemiology, National Cancer Institute, Vilnius, Lithuania Department of Consulting Clinic, National Cancer Institute, Vilnius, Lithuania; Department of Consulting Clinic, National Cancer Institute, Vilnius, Lithuania Faculty of Medicine, Vilnius University, Vilnius, Lithuania; Clinics of Rheumatology, Traumatology-Orthopaedics and Reconstructive Surgery, Faculty of Medicine, Vilnius University; Laboratory for Demographic Data, Max Planck Institute for Demographic Research, Rostock, Germany Demographic Research Centre, Vytautas Magnus University, Kaunas, Lithuania; Laboratory of Clinical Oncology, National Cancer Institute, Vilnius, Lithuania; Laboratory of Cancer Epidemiology, National Cancer Institute, Vilnius, Lithuania Institute of Public Health, Faculty of Medicine, Vilnius University, Vilnius, Lithuania

**Keywords:** breast cancer risk, parity, number of children, age at childbirth

## Abstract

**Abstract. Background.:**

Although the relationship between reproductive factors and breast cancer is internationally proved, reliable data on former USSR countries are scarce. This study examines the association of parity, age at the first childbirth, number of children, and breast cancer risk in Lithuanian women.

**Methods.:**

The study that included women from 40 to 79 years old was based on a dataset that was made up linking all records from the 2001 census, all cancer incidence records from the Lithuanian Cancer Registry and all death records from Statistics Lithuania between 6th April 2001 and 31st December 2009. Cox’s proportional hazards regression models were used to estimate the hazard ratios (HRs) for parity, age at the first childbirth, and number of children.

**Results.:**

If compared to nulliparous women, parous women had a lower risk of breast cancer (HR=0.84, 95% CI 0.78–0.89) and this risk further decreased with an increasing number of children. Women who gave birth after the age of 25 had a significantly higher risk of breast cancer. This disadvantage became statistically insignificant or decreased after controlling for total number of children.

**Conclusions.:**

Parity and age at the first childbirth are strong predictors of breast cancer risk among Lithu-anian women.

## Abstract (lt)

**Reprodukciniai veiksniai ir Lietuvos moterų krūties vėžio rizika: populiacinis kohortos tyrimas:**

**Santrauka.**

Nors tarptautiniu mastu įrodytas reprodukcinių veiksnių ir krūties vėžio ryšys, trūksta patikimų duomenų iš buvusių Sovietų sąjungos šalių. Šiame tyrime nagrinėjamas Lietuvos moterų gimdymų, amžiaus pirmojo gimdymo metu, vaikų skaičiaus bei krūties vėžio ryšys.

**Medžiaga ir metodai.**

Į tyrimą įtrauktos 40–79 metų amžiaus moterys. Tyrimo tikslu buvo sudaryta duomenų bazė, sujungianti visus 2001 m. gyventojų surašymo įrašus, visus Lietuvos vėžio registro duomenis apie vėžį ir visus Statistikos departamento mirčių duomenis nuo 2001 m. balandžio 6 d. iki 2009 m. gruodžio 31 d. Siekiant įvertinti gimdymų, amžiaus pirmojo gimdymo metu ir vaikų skaičiaus rizikos santykį, pritaikyta Cox regresijos analizė.

**Rezultatai.**

Palyginti su negimdžiusiomis moterimis, gimdžiusių moterų krūties vėžio rizika buvo mažesnė (SR = 0,84, 95% PI 0,78–0,89) ir ši rizika dar labiau sumažėjo didėjant vaikų skaičiui. Moterims, kurios pagimdė po 25 metų amžiaus, krūties vėžio rizika buvo kur kas didesnė. Atlikus koregavimą pagal bendrą vaikų skaičių, ši neigiama tendencija tapo statistiškai nereikšminga arba sumažėjo.

**Išvados.**

Gimdymas ir amžius gimdymo metu yra svarbūs predikciniai krūties vėžio rizikos veiksniai.

**Raktažodžiai:** krūties vėžio rizika, gimdymai, vaikų skaičius, amžius gimdymo metu

## Background

Reproductive and hormonal factors play an important role in breast cancer [1,2]. There is ample epidemiologic evidence showing that reproductive history such as early menarche, late age at the first childbirth, low parity, nulliparity, shorter period of breast-feeding, and older age at natural menopause are associated with an increased risk of both premenopausal and post-menopausal breast cancer [1,3-6]. A number of studies concerning the association of reproductive history and breast cancer risk, have reported that parity, early first childbirth, and an increasing number of full-term births are associated with a reduced long-term breast cancer risk [2,7-12]. However, the existing evidence about the relationship between reproductive history and breast cancer is restricted to Western countries, whereas much less is known about the corresponding associations in other regions including Central and Eastern Europe. Since the beginning of the 1990s, Lithuania has been undergoing significant transformations in family life and a decline in fertility, and the impact of these factors on breast cancer risk needs evaluation [13]. Based on a unique, regional, census-linked cancer registry data for Lithuania, this study contributes to the scientific knowledge by systematically examining the association of parity, age at the first childbirth, number of children, and breast cancer risk in Lithuanian women.

## Methods

The study was based on a census-linked dataset based on linkages between all records from the 2001 population Census, all cancer incidence records from the Lithuanian Cancer Registry, and all death and emigration records from Statistics Lithuania for the period between 6 April 2001 and 31 December 2009. Linkages between the census, emigration, death, and Cancer Registry records were implemented using personal identification numbers as unique identifiers of individuals. For individuals who died or emigrated, the exposure time was censored at the date of death or emigration. All of the linkage procedures were implemented by employees of Statistics Lithuania, who have permission to work with individual level data. Only aggregated data in a frequency table format that combines aggregated cancer cases, deaths, and population exposures for every possible combination of relevant socio-demographic and epidemiological variables were provided for this study. As this is a population-based study, members of the research team did not have access to the individual-level data containing identifiers. No special administrative permissions were required to access the aggregated anonymized census-linked dataset.  This study did not use any individual data containing personal identifiers.

For the analysis, the frequency records representing two or more cases were split into individual records. 

The study population included all women aged 40–79 in the 2001 census. Information about parity, age at the first childbirth, and number of children was taken from the census. Women with missing information about parity and/or number of children were excluded from the study (1.5%). Data about diagnosed breast cancer was taken from the Cancer Registry records. Women were observed from 6th of April 2001 until the date of breast cancer diagnosis, death, emigration, or the end of follow up (December 31, 2009), whichever came first. 

The Cox proportional hazard regression model, with person-years of follow-up as the time scale, was used to estimate the hazard ratios (HRs) and corresponding 95% confidence intervals (CIs) of developing breast cancer. The final multivariate Cox model was adjusted for parity, age at the first childbirth, and number of children. Effects of parity were estimated using the data for all women, whereas the remaining analyses (including multivariate models) were restricted only for women with children. Age at the census date was treated as a continuous variable. STATA 11 statistical software was used to carry out the analyses.

## Results

Table 1 illustrates selected characteristics of the study population. 

**Table 1.** Characteristics of the study population

Characteristics		N	%
Nulliparous		80598	9.8
Number of children	1	171802	21.0
	2	355156	43.4
	3	129091	15.8
	4	44087	5.4
	5+	38488	4.7
Age at the first childbirth	< 20	50549	6.8
	20-24	356520	48.3
	25-29	225183	30.5
	30 +	102689	13.9
	Unknown	3683	0.5
Age at census	40-49	249380	30.4
	50-59	206852	25.2
	60-69	204597	25.0
	70-79	158393	19.3

In total, the dataset includes 819 222 women, which were followed for breast cancer, deaths, and emigration for an average 8.6 years. During the period of observation, a total number of 9 711 new breast cancer cases were diagnosed, whereas the total number of women person-years of exposure to risk amounted to 6.6 million. 

The univariate results suggest that nulliparous status is strongly associated with breast cancer (Table 2). 

**Table 2.** Hazard ratios (HR) of breast carcinoma according to reproductive variables

Characteristics		HR (95 % CI)						
		Cancers	Univariate			Multivariate^**^		
Parity	Nulliparous	1081	ref.	-	-	-	-	-
	Parous	8630	0.84	0.78	0.89	-	-	-
Age at the first childbirth^*^	< 20	513	ref.	-	-	ref.	-	-
	20-24	3898	1.05	0.96	1.16	1.00	0.91	1.09
	25-29	2734	1.18	1.08	1.30	1.04	0.94	1.14
	30 +	1449	1.40	1.26	1.55	1.14	1.03	1.26
Number of children	1	2298	ref.	-	-	ref.	-	-
	2	4332	0.90	0.85	0.94	0.93	0.88	0.97
	3	1340	0.78	0.73	0.83	0.78	0.72	0.83
	4	363	0.63	0.57	0.71	0.62	0.55	0.69
	5+	297	0.60	0.54	0.68	0.58	0.51	0.65

^*^ for 36 cases, age was not reported

^**^ adjusted for age at the first childbirth, and number of children

If compared to nulliparous women, parous women had 16% lower risk of breast cancer (HR=0.84, 95% CI 0.78–0.89) and the risk of breast cancer further decreased with increasing number of children. Age at the first childbirth shows a significant risk only for women who gave birth after age of 30 (HR=1.40, 95% CI 1.26–1.55). However, in the multivariate analysis after adjustment for number of children, the disadvantage of women having a first child after the age of 30 notably decreased (HR=1.14, 95% CI 1.03–1.26). With increasing number of children, the risk of breast cancer was decreasing even after adjustment for age at first childbirth.

## Discussion

The differences of breast cancer incidence in Europe are likely due to variations in reproductive factors, such as postponed first childbirth and lower parity across populations, and these changes in the childbearing pattern during recent decades will probably affect cancer incidence in the future [14]. Breast cancer is the most frequent cancer among females in Lithuania and the incidence rate has been steadily increasing during the last few decades [15]. This coincides with a notable change in childbearing patterns such as an increase in the late first births after the age of 30 [13]. 

Despite very rapid changes in childbearing patterns during the last two decades, the results of our study generally correspond to those observed in other European countries. A French prospective cohort study using the data of 1718 breast cancer cases revealed the protective effect of high parity on breast cancers [4]. A meta-analysis of 8 studies in Nordic countries including a total of 5 568 cases, confirmed a 30% increase in breast cancer risk in nulliparous women compared with parous women, and for every 2 births, the risk was reduced by about 16% [5]. Another meta-analysis based on 20 studies, determined that each child led to a 3% reduction in the risk of premenopausal breast cancer, whereas the reduction reached 12% for postmenopausal breast cancers [6]. Even in a low-risk population of Japanese women aged 40-69 years, nulliparity and low parity were significantly associated with an increased risk of breast cancer [12]. Data from comparative study conducted in Estonia and Slovakia also confirmed such association. Relative risk (RR) for estimates in the nul-liparous were 1.70 (0.94-3.07) in Estonian and 2.69 (1.31-5.51) in Slovak women. Estonian women had the lowest RR for breast cancer after birth of the second child. In women form Slovakia the RR decreased successively with increasing parity [16].

Our results suggest that the risk of breast cancer decreased in parous women with an increasing number of children and this advantage persisted after adjustment for the age at the first childbirth. Meta-analysis of three large case-control studies of breast cancer with a total data set of 4 072 cases and 4,099 controls from several Italian regions, identified that the breast cancer risk after adjustment for age at first live birth, did not differ among women with one to four live births, but was significantly below unity (relative risk (RR) 0.6) for those with five or more live births [3]. The large Finnish nationwide cohort study of 86 978 women with at least five births, reported that a higher number of births was an independent protective factor against breast cancer [8]. A markedly decreased breast cancer risk was also reported in Finnish women with at least 10 childbirths [11].

Having the first childbirth at the age of 30 or older, was a significant risk factor for breast cancer in the present study. Meta-analysis of Negri et al. also ascertained that the breast cancer risk was directly related to age at the first live birth (RR 1.8 for ≥28 years versus < 22 years) [3]. In the previously mentioned study in Estonia and Slovakia the breast cancer risk was the lowest for the women who gave birth for the first child before the age of 20 years [16]. Ewertz et al., observed a significant trend of increasing risk with older age at the first childbirth, women giving the first birth after the age of 35 years having a 40% increased risk compared to those with the first childbirth before the age of 20 years [5]. Late first full-term pregnancy (above age 30 years) conveyed a risk of 1.63 (95% CI 1.12–2.38) and 1.35 (95% CI 1.02–1.78) in pre- and postmenopausal groups, respectively [4]. Breast cancer risk increased with increasing age at the first birth by 5% per year for premenopausal breast cancer and by 3% for cancers diagnosed late or after menopause [6]. Rhode Island (USA) cohort data showed that the estimated first birth median age of 41 years carried a significantly increased relative risk of 3.7 (95% CI 1.30–10.5) compared with a baseline group with the age of 23. The risk of breast cancer increased by 8% with each passing year after the first birth [7]. A Norwegian prospective study, including in total 23890 women, showed that the increase in the mother’s age among multiparous women influenced the long-term breast cancer risk level [9]. 

An analysis of Finnish grand multiparous women data showed that an older age at the first childbirth (≥ 30 years versus <20 years) nearly doubled the breast cancer risk (from 0.40 to 0.73) [8]. A Swedish cohort study showed that the relative breast cancer risk was 0.35 (95% CI 0.30–0.42) for women with 6 or more children and the first child before the age of 20 years; and the risk was 1.11 (95% CI 1.06–1.18) for women with the first birth at age 35 years or older, compared to nulliparous women [10].

The methodological strengths of this study include a cohort design based on the follow-up of the entire population of Lithuania, a large sample size, a substantial number of women with breast cancer and a reliable identification of the cases over the study period. During the period of observation, women were followed for breast cancer, deaths, and emigration for an average 8.6 years, whereas the total number of person-years of exposure amounted to 6.6 million. The main limitation of the study is the retrospective nature; therefore, we could not take into account exposure confounding from other hormonal and reproductive factors which may have influenced the results (miscarriages and abortions, stillbirths, hormone use, breastfeeding, etc.).

## Conclusions

Our findings suggest that parity, age at the first childbirth, and number of children are important risk factors of breast cancer among Lithuanian women, however, a further more in-depth research is needed to examine potentially important direct and confounding effects of socio-economic and biomedical variables.
